# Early intervention with acupuncture improves the outcome of patients with Bell's palsy: A propensity score-matching analysis

**DOI:** 10.3389/fneur.2022.943453

**Published:** 2022-09-14

**Authors:** Lian-Sheng Yang, Dan-Feng Zhou, Shu-Zhen Zheng, Bai-Ming Zhao, Huo-Gui Li, Qi-Qing Chen, Yun Zhong, Hong-Zhi Yang, Kun Zhang, Chun-Zhi Tang

**Affiliations:** ^1^Guangzhou University of Chinese Medicine, Guangzhou, China; ^2^Department of Acupuncture and Moxibustion, The Third Affiliated Hospital of Sun Yat-Sen University, Guangzhou, China; ^3^Department of Traditional Chinese Medicine, The Third Affiliated Hospital of Sun Yat-Sen University, Guangzhou, China; ^4^South China Research Center for Acupuncture and Moxibustion, Medical College of Acu-Moxi and Rehabilitation, Guangzhou University of Chinese Medicine, Guangzhou, China

**Keywords:** acupuncture, Bell's palsy, real world study, propensity score matching, retrospective study

## Abstract

**Objective:**

Although acupuncture is widely used as a complementary therapy in the treatment of Bell's palsy (BP) when to initiate acupuncture is still controversial. This study aims to determine the efficacy of the early intervention by acupuncture on BP.

**Methods:**

We retrospectively gathered clinical data from the Third Affiliated Hospital of SUN-YAT SEN University between 2016 and 2021. We selected newly diagnosed patients with BP who were diagnosed by registered neurologists or acupuncturists formally. The qualified patients were divided into two groups according to whether or not initial acupuncture treatment was given within 7 days from the onset of palsy. Cohorts were balanced using 1:1 propensity score matching (PSM). Cox proportional hazards modeling and Kaplan–Meier analysis were applied to determine the differences between the two groups. The outcome included time to complete recovery of facial function, the rate of complete recovery, and the occurrence of sequelae in 24 weeks.

**Results:**

A total of 345 patients were eligible for this study and were divided into the manual acupuncture/electroacupuncture (MA/EA) group (*n* = 76) and the EA group (*n* = 125). In the propensity score-matched cohort, the time to complete recovery was significantly shorter in the MA/EA group compared with the patients in the EA group (hazard ratio 1.505, 95% CI 1.028–2.404, *p* <0.05). The MA/EA group had a higher rate of favorable outcomes at 12 weeks than the EA group (93.4 vs. 80.3%, *p* = 0.032), and the occurrence of sequelae at 24 weeks showed a greater reducing trend in the MA/EA group than the EA group (6.6 vs. 16.4%, *p* = 0.088).

**Conclusion:**

Acupuncture intervention at the acute stage of BP could shorten the time to recovery and improve the outcome.

**Clinical trial registration:**

http://www.chictr.org.cn, identifier ChiCTR 2200058060.

## Introduction

Bell's palsy (BP) is a common cranial mononeuropathy that accounts for 70% of peripheral facial palsies. It presents as unilateral weakness or paralysis of the face due to acute dysfunction of the peripheral facial nerve with no readily identifiable cause (1).

The described population incidence rates range from 11.5 to 40.2/100,000 (2, 3). Approximately, 30% of patients with BP have sequelae that include residual paresis, contracture, and synkinesis, which have a dramatic impact on social function, emotional expression, and psychological health (4, 5).

Previous randomized-controlled studies demonstrated the improving effect of corticosteroids on treating BP (6, 7). Moreover, it showed a greater benefit of using corticosteroids in the acute stage (8). The effect of antiviral drugs is still inconclusive (9, 10). Predictors of incomplete recovery were old age, severe facial palsy, no recovery in the first 3 months after onset, prolonged pain around the ear, pregnancy, diabetes mellitus, and hypertension. However, medications exhibit limited efficacy to promote neurological function recovery. To minimize the time to recovery and sequelae from BP, the most effective treatment has to be established, including medication and non-pharmacological therapy.

As an adjuvant therapy, acupuncture has been widely applied to neuropathy throughout East Asia for more than 4,000 years. Numerous clinical trials have shown that acupuncture could improve facial motor symptoms, release pain around the ear, and speed up recovery (11, 12). However, when to initiate the intervention by acupuncture is still controversial (13). Evidence is scarce regarding whether acupuncture in the acute stage within 7 days of palsy onset could improve the outcome of BP in long-term follow-up. Therefore, to address this gap, we aim to investigate the effect of acupuncture therapy initiating in the acute stage of BP based on relevant real-world data.

## Materials and methods

### Participants

In this single-center retrospective study, a total of 345 patients who underwent acupuncture secondary to BP between August 2016 and December 2021 were enrolled. The inclusion criteria were as follows: (1) patients with unilateral facial paralysis, (2) patients with neurological deficits with a House–Brackmann (H-B) grading of IV or higher during treatment, (3) patients ≥18 years of age, and (4) patients who underwent acupuncture and follow-up in the outpatient department.

The exclusion criteria were as follows: (1) facial nerve dysfunction caused by herpes zoster virus; (2) otogenic facial paralysis such as otitis media, labyrinthitis, and mastoiditis; (3) peripheral facial paralysis secondary to brainstem injury, acoustic neuroma, Guillain–Barre syndrome, or other neurological diseases; and (4) incomplete data or loss to follow-up.

This study was approved by the Medical Ethics Committee of the Third Affiliated Hospital of SUN YAT-SEN University. The procedures of this study adhered to the tenets of the Declaration of Helsinki. This study was registered in the Chinese Clinical Trial Registry (ChiCTR 2200058060) on 28 March 2022.

### Interventional procedures

All patients received standard medication management according to the latest Chinese Guidelines for BP (14). Prednisolone 30 mg was given daily orally for 7 days, and the dose was then reduced by 10 mg per day for 7 days, with a total treatment time of 21 days. In addition, acupuncture treatment based on the theory of Traditional Chinese Medicine was performed on all patients with BP. We adopted differentiated approaches according to the different timing of intervention of acupuncture. The details of acupuncture such as the chosen acupoints and needling methods are provided in the Additional File 1. The therapy was carried out by licensed acupuncturists having more than 5 years of experience.

Patients with onset of BP within 7 days received mildly manual acupuncture (MA) therapy. Sterile, stainless steel needles (length: 25 mm; diameter: 0.3 mm; Sui Xin, Suzhou Medical Appliance, Suzhou, China) were inserted into the described acupoints. After patients indicated the *Deqi* sensation (soreness, heaviness, and distension sensation), needles were left in acupoints, and manipulations of twirling, lifting, and thrusting were performed once every 10 min. The treatment was given for 30 min during each session, one time per day within 7 days after onset. After accepting MA, the patients with onset beyond 7 days would continue to receive electroacupuncture (EA) therapy.

When palsy onset was beyond 7 days, we initiated regular EA therapy in patients. After needles were inserted into acupoints, paired alligator clips from an EA apparatus (GB6805-2, Medical Supply & Equipment Co, Ltd, Shanghai, China) were attached transversely to the needle-holders. EA stimulation lasted for 30 min with a continuous wave of 20 Hz frequency, a pulse width of 0.5 ms, and a current intensity of 0.1–2 mA depending on the individual participant's comfort level (preferably with the skin around the acupoints shivering mildly without pain). The EA was administered three times per week for 24 weeks or until complete recovery.

### Follow-up

Patients were followed until recovered completely. If recovery was incomplete within 6 weeks, the next follow-up was at 12 and 24 weeks. The facial function was assessed at all visits with the H-B scale. The H-B scale is a grading system based on a six-grade score, where I is a normal function and VI is complete paralysis, for gross assessment of facial motor function and sequelae.

### Data collection and outcome measures

Detailed clinical information was extracted through a retrospective review of the patient record, procedure notes, and follow-up notes. Baseline characteristics were collected, including age, gender, symptomatic presentation, previous history of BP, perinatal period, comorbidities (hypertension, diabetes mellitus, hyperlipidemia, and psychiatric disease), onset time, time from the onset to medical interventions, and H-B grading before acupuncture. We considered the primary outcome was time to complete recovery of facial function, defined as an H-B grading of I. The secondary outcome was the rate of complete recovery and the occurrence of synkinesis, facial spasm, or contracture.

### Statistical analysis

We used mean and SD for normally distributed variables, or median (interquartile) for the variables not normally distributed, to summarize the participants' demographics. Statistical analyses were performed using SPSS version 26.0. Comparisons were performed using the *t*-test or Mann–Whitney test for continuous variables and chi-square test for categorical variables, as appropriate. All tests were two-sided, and an α <0.05 was considered significant.

Propensity score matching (PSM) was performed using SPSS version 26.0 to ensure an even distribution of possible confounders between the two groups. A 1:1 matching range by using proximity matching was performed with a caliper width of 0.01. The underlying characteristics considered in the propensity-matching process were age, gender, hypertension, diabetes mellitus, hyperlipidemia, psychiatric disease, perinatal period, history of BP, and time from the onset to medical interventions. After matching patient characteristics, the Kaplan–Meier method was used to estimate survival curves. Cox proportional hazards models were used to estimate the hazard ratio (HR) of recovery and the corresponding 95% confidence interval (CI).

## Results

### Demographic characteristics between two groups

**Figure 1:**
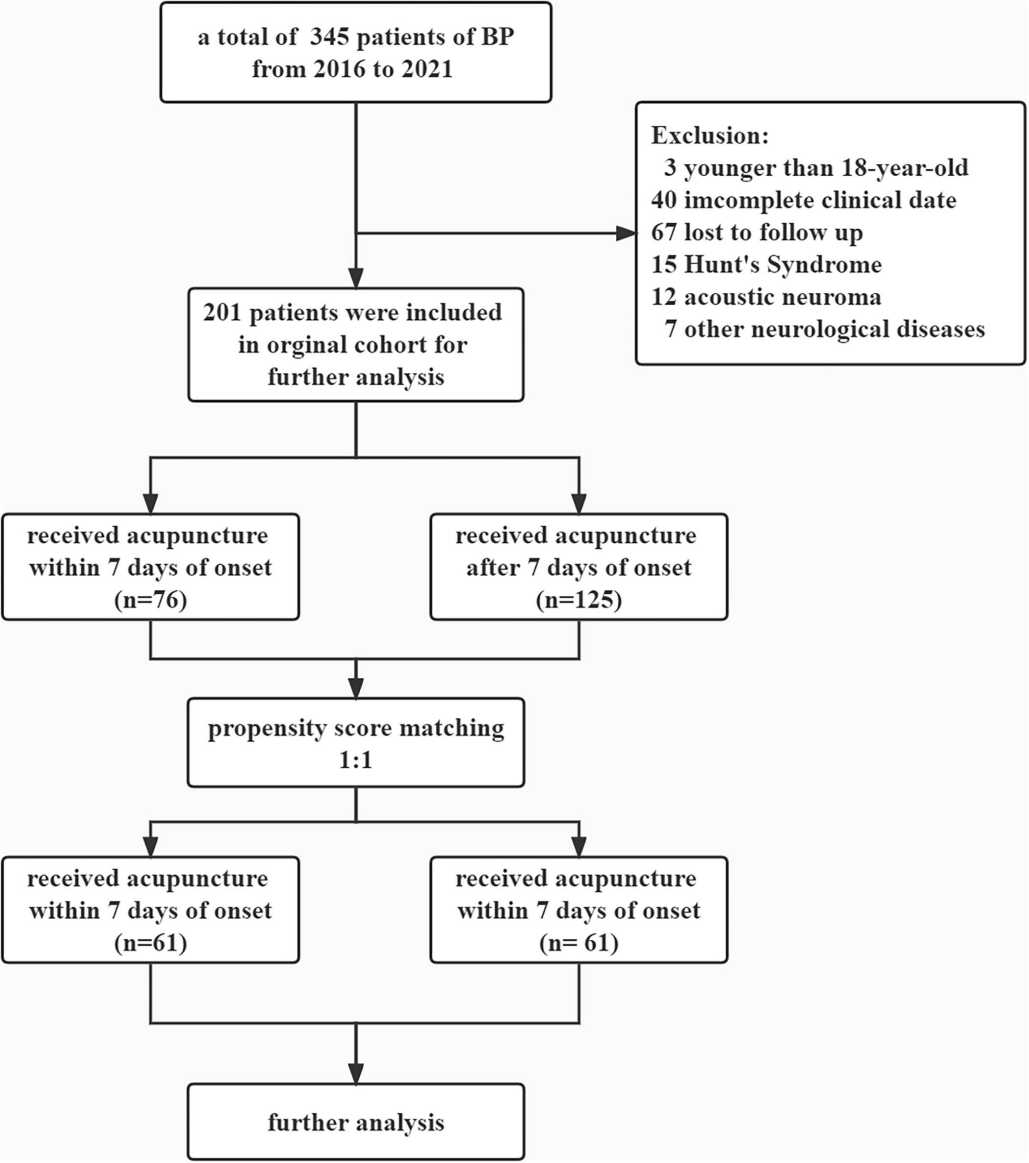
Flowchart of the included participants.

The flowchart is summarized in Figure 1. A total of 345 patients with newly diagnosed BP were qualified for the study. Among the included subjects, 76 (37.8 %) patients were treated with acupuncture within 7 days after onset, and 125 patients (62.2%) received acupuncture therapy over 7 days after onset (Table 1). Significant differences between the MA/EA group and the EA group were observed. Specifically, the characteristics, namely, age, medical history, and comorbidities between the two groups are statistically significant (*p* <0.05). Patients in the MA/EA group were more likely to seek treatment actively (80.3 vs. 65.6%, *p* = 0.026).

**Table 1:** Characteristics of patients before and after propensity score matching.

	**Before PSM**				**After PSM**			
**Variable**	**MA/EA** **(*n =* 76)**	**EA** **(*n =* 125)**	***P*-Value**	**Standardized** **Difference, %**	**MA/EA** **(*n =* 61)**	**EA** **(*n =* 61)**	***P*-Value**	**Standardized** **Difference, %**
Age	34.56 ± 12.61	39.24 ± 14.38	***0.020***	34.6	34.33 ± 11.49	35.36 ± 12.16	0.655	8.7
**Gender**
Female	39 (51.3)	61 (48.8)	0.729	5.0	31 (50.8)	31 (50.8)	1.000	0
Male	37 (48.7)	64 (51.2)		5.0	30 (49.2)	30 (49.2)		0
**Presentation**
Numb in Tongue	8 (10.5)	15 (12.0)	0.750	4.7	6 (9.8)	7 (11.4)	0.769	5.2
Pain around the ear	36 (47.3)	64 (51.2)	0.598	7.8	18 (29.5)	20 (32.7)	0.695	6.9
Hyperacusis	3 (3.9)	4 (3.2)	0.752	3.7	1 (1.6)	1 (1.6)	1	0
History of BP	4 (5.3)	1 (0.8)	***0.049***	26.3	1 (1.6)	1 (1.6)	1.000	0
**H-B facial grading**
IV	23 (30.3)	38 (30.4)	0.944	0.2	17 (27.9)	21 (34.4)	0.733	14.1
V	49 (64.5)	79 (63.2)		2.7	42 (68.9)	38 (62.3)		13.9
VI	4 (5.3)	8 (6.4)		4.7	2 (3.3)	2 (3.3)		0
Perinatal period	1 (1.3)	6 (4.8)	0.191	0	1 (1.6)	1 (1.6)	1.000	0
**Baseline comorbidity**
Diabetes mellitus	3 (3.9)	12 (9.6)	0.139	22.9	3 (4.9)	4 (6.3)	0.697	8.9
Hypertension	0 (0)	9 (7.2)	***0.017***	39.4	0 (0)	0 (0)	/	0
Hyperlipidemia	2 (2.6)	4 (3.2)	0.818	3.57	0 (0)	0 (0)	/	0
Psychiatric disease	2 (2.6)	2 (1.6)	0.612	6.9	1 (1.6)	1 (1.6)	1.000	0
**Corticosteroid therapy**
Within 72 h	61 (80.3)	82 (65.6)	***0.026***	33.6	46 (75.4)	45 (73.8)	0.835	3.7

We ultimately used a 1:1 PSM method to select 61 patients in each group, respectively, in order to minimize the interference of confounding variables. The patients in the two groups were matched on the basis of similarities in their demographics, comorbidities, medicines used, and the time from the onset of palsy to medications, and all covariates were statistically indistinguishable between the two groups. After the matching procedure, there were no significant differences in gender, age, medical history, baseline comorbidities, and time from the onset of palsy to the start of medication management between the two groups. The median durations between the onset of palsy and the first receiving of acupuncture in the two groups were 3 and 9 days, respectively.

### The effect of acupuncture within 7 days after onset of palsy

**Figure 2:**
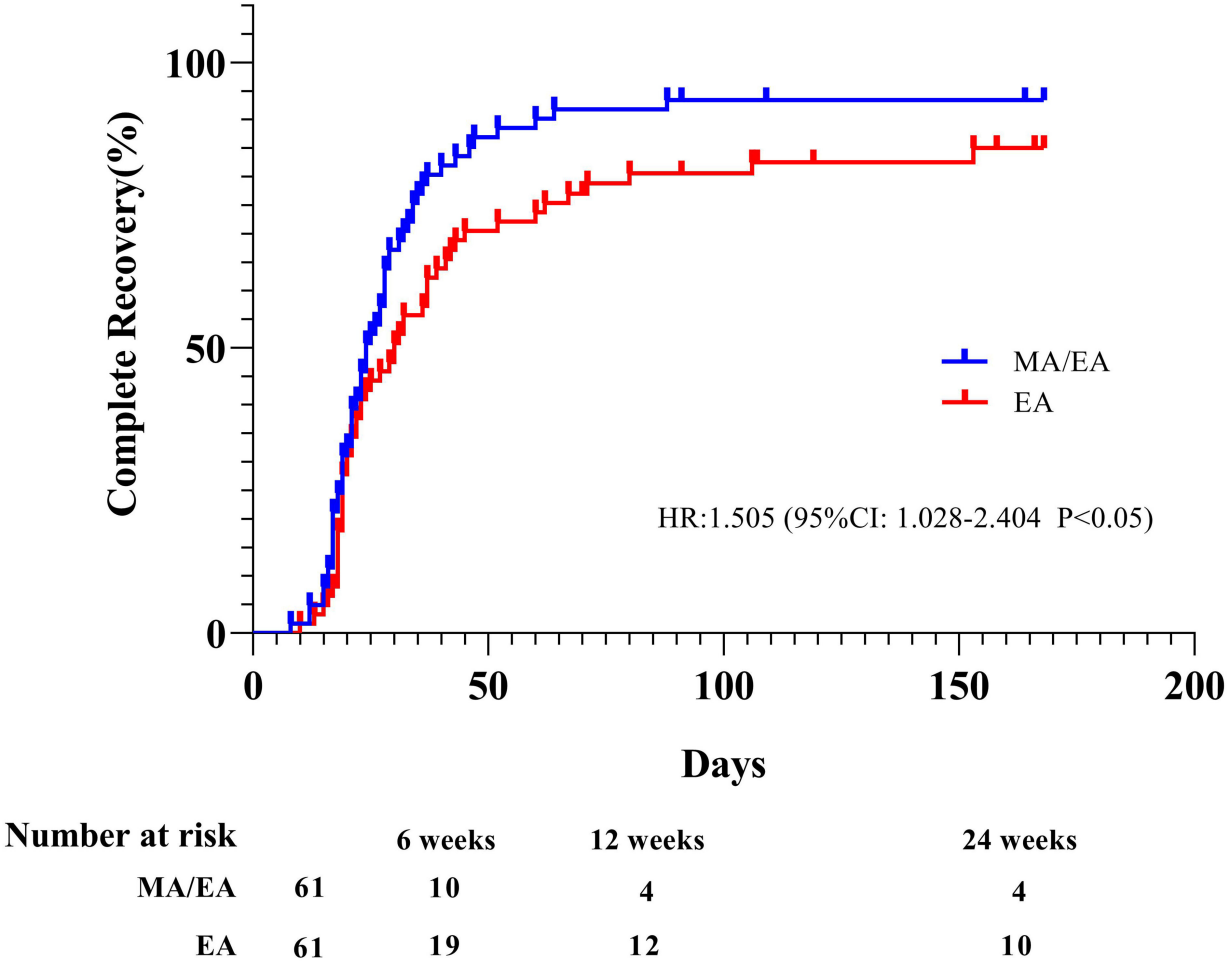
Kaplan–Meier estimates of patients who made a complete recovery. House–Brackmann (H-B) grading of I in the two groups (*n* = 122). The Kaplan–Meier estimates are based on the actual number of days from the onset of palsy to the first follow-up visit when the patient had completely recovered.

Figure 2 shows that the time to complete recovery was significantly shorter for the patients who received acupuncture within 7 days compared with the patients treated over 7 days (HR 1.505, 95% CI 1.028–2.404, *p* < 0.05). The mean time to complete recovery (H-B grading of I) in the MA/EA group was shorter than in the EA group (36.62 ± 4.78 vs. 55.40 ± 6.93, *p* < 0.05).

Table 2 shows that the patients in the MA/EA group had significantly higher recovery rates at 12 weeks than the patients in the EA group (*p* < 0.05). At 12 weeks, 57 of 61 patients (93.4%) in the MA/EA group had recovered compared with 49 of 61 patients (80.3%) in the EA group.

**Table 2:** Comparison of rates of complete recovery per follow-up visit between two groups.

**Groups**	**6 weeks (*n =* 61)**	**12 weeks (*n =* 61)**	**24 weeks (*n =* 61)**
MA/EA group	51 (83.6)	57 (93.4)	57 (93.4)
EA group	42 (68.9)	49 (80.3)	51 (83.6)
*P*-Value	0.056	***0.032***	0.088

Of the 122 patients who had 24 weeks' follow-up, sequelae were reported in 4 of 61 patients (6.6%) treated within 7 days compared with 10 of 61 (16.4%) in those who did not receive acupuncture within 7 days. But these trends in the difference were not significant statistically (*p* > 0.05) (Table 3).

**Table 3:** Comparison of occurrence of synkinesis between two groups.

**Groups**	**Occurrence of synkinesis at 24 weeks (*n =* 61)**
MA/EA group	4 (6.6)
EA group	10 (16.4)
*P*-Value	0.088

Acupuncture-related adverse events (AEs) occurred in 4/61(6.6%) participants in the MA/EA group and 2/61(3.3%) in the EA group (*p* > 0.05). The most commonly reported acupuncture-related AEs included subcutaneous hemorrhage, sharp pain after acupuncture, and fainting during treatment (Table 4). No significant difference was found between the two groups for the proportion of patients with AEs (*p* > 0.05 for all AEs). Neither group had severe AEs. None of the participants withdrew from the study because of AEs.

**Table 4:** Adverse events related to acupuncture between two groups^a^.

**Adverse event**	**MA/EA (*n =* 61)**	**EA (*n =* 61)**	***P*-Value**
Overall	4	2	0.402
Subcutaneous hemorrhage	2	1	0.558
Sharp pain after acupuncture	1	1	/
Faint during acupuncture	1	0	0.315
infection around the site of needling	0	0	/

^a^Adverse events were counted by type rather than frequency in the same participant. Adverse events with different types occurring in a single participant were defined as independent adverse events. An adverse event with multiple occurrences in a single participant was defined as 1 adverse event.

## Discussion

In this PSM-based real-world study, we evaluated the efficacy of the early intervention by acupuncture therapy in patients with BP. The main findings of our study are as follows: (1) patients in the MA/EA group had a shorter time to complete recovery, and clinical outcomes at 12 weeks were more favorable in these patients than in those who did not receive acupuncture in the acute phase and (2) the effect of acupuncture in the acute phase of BP showed a trend that was better than those who did not on reducing the occurrence of sequelae. Larger sample sizes would be needed to show whether these trends in the difference between the two groups may be statistically significant.

Over the years, whether to perform acupuncture in the acute phase of BP has been hugely inconclusive and debatable (15). The focus of this issue lies in the time window of acupuncture and the method of stimulation. Some studies have revealed that an early and appropriate acupuncture therapy might shorten the time to recovery, whereas others insist that EA is not appropriate for patients with BP in the acute phase because it is inclined to aggravate edema and bioelectric conduction disorders of the facial nerve (16, 17). Based on this controversy, we adopted the stimulation of mild MA cautiously to avoid the potential hazards to patients with BP. This study provides evidence for the procedure and strategy of intervention by acupuncture in the acute phase of BP. In addition, the proportions of participants having acupuncture-related AEs in the MA/EA and EA groups were low, and the specific AEs were mild or transient. These results are similar to those reported in a previous acupuncture study (18).

A previous study by Engström M and coworkers showed that the complete recovery rates were ~70% in patients with BP receiving prednisolone (6). A study found that ~70% of patients with BP recover completely within 6 months without treatment (5). In this study, our recovery rates were higher than those previously reported. The causes of the differences between this study and the previous study might be the lower recovery rates assessed by the Sunnybrook scale vs. the H-B scale. Besides, we cautiously attribute the difference as an additional benefit of acupuncture intervention plus corticosteroids probably.

Although it is reported that the Sunnybrook system assesses facial function continuously, has a wider response range, and is more reliable than the H-B scale, we used the H-B scale instead of the Sunnybrook scale as the main scale to assess facial nerve function in this study. The causes were as follows: (1) evaluation with the H-B scale is easier and quicker than that with the Sunnybrook system, which is more likely to be in accordance with the current clinical situation, and (2) we focused more on endpoint evaluation, not the continuous observation of facial nerve function.

Previous findings indicate that inflammation and edema of the facial nerve are part of the pathogenesis in patients with BP (19, 20). Similar to the effect of corticosteroids on BP, the effect of acupuncture may be related to its anti-inflammatory effect. Some *in vivo* evidence suggest that acupuncture at ST 36 and ST 25 regions could produce anti-inflammatory effects by driving the vagal–adrenal axis (21). Besides, the benefit of using corticosteroids is unclear when more than 72 h have elapsed since the onset of palsy. Likewise, when beyond the time window of acupuncture, the benefit of acupuncture might decrease.

### Strengths and limitations of the study

Compared with other studies on acupuncture for BP, the protocol of this study had some advantages. (1) It was easier to execute than a prospective randomized-controlled trial (RCT). An RCT study reported that it is difficult to recruit and maintain patients (11). This protocol was more conducive to protecting patients' rights and avoiding delays in treatment owing to the study design. (2) It could avoid the bias between two groups through a variety of statistical methods. Although RCT is the best way to minimize bias in ascertaining treatment effects, many studies have reported the uneven distribution of initial facial grade even if it was grouped by randomization (11, 22). (3) We took time to recover completely as primary endpoints, which was rare in the study of acupuncture for BP, whereas it was common in the study of other treatments for BP.

This study still has some limitations. First, single-center design, high rate of loss to follow-up (67/345), and small sample size are the major limitations of this study. Second, although propensity score-based matching analysis was applied in the study to reduce bias, it still has deficits such as shrinkage of the cohort's sample size and failure to eliminate unknown bias like the potential effect of antivirus treatment and unclear benefits of corticosteroid over 3 days. Third, we did not compare groups with no acupuncture or sham acupuncture. Furthermore, studies with multicenter, large samples are still needed to prove the effect of early intervention by acupuncture on BP.

## Conclusion

This study revealed that acupuncture intervention at the acute stage of BP could shorten the time to recovery and improve the outcome. The findings support the use of acupuncture as a complementary therapy for BP in the acute phase.

## Data availability statement

The raw data supporting the conclusions of this article will be made available by the authors, without undue reservation.

## Ethics statement

The studies involving human participants were reviewed and approved by Medical Ethics Committee of Third Affiliated Hospital of Sun Yat-Sen University (Guangzhou, China). Written informed consent for participation was not required for this study in accordance with the national legislation and the institutional requirements.

## Author contributions

L-SY and KZ: study design. B-MZ, H-GL, Q-QC, and YZ: data collection. S-ZZ and D-FZ: statistical analysis. L-SY and D-FZ: draft writing. H-ZY: draft review and modification. C-ZT: result interpretation, draft review, and editing. All authors contributed to the article and approved the submitted version.

## Funding

This work was funded by the Construction Project of Inheritance Studio of National Famous and Old Traditional Chinese Medicine Experts in 2022.

## Conflict of interest

The authors declare that the research was conducted in the absence of any commercial or financial relationships that could be construed as a potential conflict of interest.

## Publisher's note

All claims expressed in this article are solely those of the authors and do not necessarily represent those of their affiliated organizations, or those of the publisher, the editors and the reviewers. Any product that may be evaluated in this article, or claim that may be made by its manufacturer, is not guaranteed or endorsed by the publisher.
