# Supplementary material for: Early intervention with acupuncture improves the outcome of patients with Bell's palsy: A propensity score-matching analysis
Source: Front Neurol. 2022 Sep 14;13:943453. doi: 10.3389/fneur.2022.943453 (PMC9517937; doi:10.3389/fneur.2022.943453)

**Supplementary Content**

| onset | acupoints | Location | Angle when inserting needle | Depth | Paired when EA |
| --- | --- | --- | --- | --- | --- |
| ≤7 days | BL2 | at the medial end of the eyebrow | 10-15 | 15 mm | / |
|  | ST1 | Directly below the pupil, and between the eyeball and infraorbital ridge | 10-15 | 15 mm | / |
|  | ST4 | Directly below the pupil, and lateral to the corner of the mouth | 10-15 | 10 mm | / |
|  | ST6 | One finger-breadth anterosuperior to the mandibular angle, at the depression anterior to the masseter where is easy to see in clenching the teeth | 10-15 | 10 mm | / |
|  | LI 20 | Lateral to the midpoint of the nasal ala, and in the nasolabial groove | 10-15 | 25 mm | / |
|  | GB20 | At the level of inferior to the external occipital protuberance, in the depression between the upper ends of the sternocleidomastoid trapezius muscles | 80-90 | 25 mm | / |
| >7 days | BL2 | See above | 10-15 | 15 mm | Paired |
|  | GB14 | Directly above the pupil, 1 cun above the midpoint of the eyebrow | 10-15 | 10 mm |  |
|  | ST1 | See above | 10-15 | 15 mm | Paired |
|  | LI 20 | See above | 10-15 | 25 mm |  |
|  | ST4 | See above | 10-15 | 10 mm | Paired |
|  | ST6 | See above | 10-15 | 10 mm |  |
|  | SI18 | Directly below the lateral canthus, the depression below the zygomatic arch | 10-15 | 10 mm | Paired |
|  | EX-HN  (Qian Zheng) | 1 *cun* anterior to the earlobe | 90 | 25 mm |  |
|  | SJ17 | posterior to the earlobe, between the mastoid process and mandible angle | 90 | 20 mm | Paired |
|  | GB20 | See above | 90 | 20 mm |  |

Table 1: the plan of acupuncture in two groups

Fig 1: the timeline in retrospective study


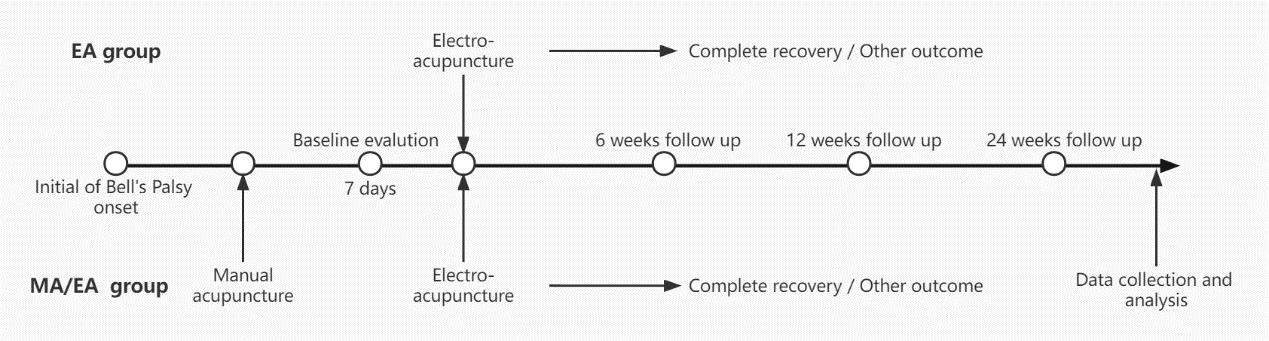

Supplement: Supplementary file 1 [file Data_Sheet_1.docx]
